# P-570. Infectious Disease Physician Office-Based HIV Program Results in Successful Adherence of Therapy in Patients Receiving Long-Acting Cabotegravir and Rilpivirine

**DOI:** 10.1093/ofid/ofae631.768

**Published:** 2025-01-29

**Authors:** Nikhil K Bhayani, Jorge R Bernett, Thomas K Sleweon, Erika M Young, Kent J Stock, Stacey E Baker, Quyen Luu, Brian S Metzger, Kimberly A Couch, Christina J Weeks, Lucinda J Van Anglen

**Affiliations:** DFW Infectious Diseases, PLLC, Bedford, Texas; Infectious Disease Doctors Medical Group, APC, Walnut Creek, California; Infectious Disease Specialists, Highland, Indiana; Infectious Disease Specialists, Highland, Indiana; Low Country Infectious Disease, Charleston, South Carolina; Infectious Disease Physicians, PA, Miami, Florida; Central Georgia Infectious Disease Associates, LLC, Macon, Georgia; Austin Infectious Disease Consultants, Austin, Texas; Healix Infusion Therapy, LLC, Sugar Land, Texas; Healix Infusion Therapy, LLC, Sugar Land, Texas; Healix Infusion Therapy, LLC, Sugar Land, Texas

## Abstract

**Background:**

The long-acting (LA) formulation of cabotegravir (CAB) and rilpivirine (RPV) demonstrated efficacy in Phase 3 studies and was approved in the US for HIV-1 in 2021. CAB+RPV LA offers treatment advantages over daily oral therapy with 1-2 month injections administered by a health care provider. However, onboarding of therapy and drug access for patients has proven challenging for physicians. We describe real-world implementation of a standardized Infectious Disease (ID) office-based CAB+RPV LA program with evaluation of adherence and virologic effectiveness.

**Methods:**

The ID in-office CAB+RPV LA program was implemented in July 2023 in a national network of ID practices. The program involved creation of standardization for drug orders and approval, nurse training and appointment management. The first 100 patients treated in the program were followed. Data included demographics, prior therapy, baseline laboratory data, adherence to schedule and follow-up laboratory indices. Adherence was defined as injections received +/- 7 days from target date.

**Results:**

Overall, 100 patients have entered the program in 20 practices nationally from July 2023 to March 2024. Median age is 49 years (IQR: 49-59), 81% male. Median weight and BMI are 89.8 kg (IQR: 80.8-101.6) and 29.2 kg/m^2^ (IQR: 27.1-31.9), respectively. Median duration of HIV disease is 7 years (IQR 4, 13) and patients received 1 (IQR: 1-2) oral regimen prior to CAB+RPV LA. A total of 55 patients initiated CAB+RPV LA in the ID physician office and 45 transferred care to their ID physician office. Median baseline viral load prior to any CAB+RPV LA was 30 (IQR: 21-65) and CD4 count was 761 (IQR: 535-1008). The majority (98%) are receiving injections every 2 months. Of 274 injections administered to date, 269 (98.2%) have been adherent to schedule. Fourteen patients eligible for 6 month follow-up labs had viral load undetectable (86%, n=12) or suppressed (14%, n=2). Six patients discontinued therapy, primarily due to financial or payor issues.

**Conclusion:**

An ID physician office-based CAB+RPV LA program resulted in high levels of injection adherence. Patients eligible for follow-up achieved a 100% level of virologic control. These results suggest that CAB+RPB LA administered through a standardized ID office-based program is effective in managing HIV-1.

**Disclosures:**

**Nikhil K. Bhayani, MD, FIDSA**, CorMedix: Advisor/Consultant|Cumberland Pharmaceuticals: Advisor/Consultant|Melinta: Advisor/Consultant|Paratek Pharmaceuticals: Advisor/Consultant **Erika M. Young, DO**, Cumberland Pharmaceuticals: Advisor/Consultant **Brian S. Metzger, MD, MPH**, Cumberland Pharmaceuticals: Advisor/Consultant|Ferring Pharmaceuticals: Advisor/Consultant **Lucinda J. Van Anglen, PharmD**, Cumberland Pharmaceuticals: Grant/Research Support|Ferring Pharmaceuticals: Grant/Research Support|Novartis Pharmaceuticals: Grant/Research Support|Takeda Pharmaceuticals: Grant/Research Support

